# The Regulatory Effects and the Signaling Pathways of Natural Bioactive Compounds on Ferroptosis

**DOI:** 10.3390/foods10122952

**Published:** 2021-12-01

**Authors:** Shenshen Zhang, Ruizhe Hu, Yaping Geng, Ke Chen, Ling Wang, Mustapha Umar Imam

**Affiliations:** 1 School of Public Health, School of Physical Education (Main Campus), Zhengzhou University, No. 100 Science Avenue, Zhengzhou 450001, China; huruizhe1618@163.com (R.H.); 17862814151@163.com (Y.G.); ck15708917322@163.com (K.C.); lisalingwang@zzu.edu.cn (L.W.); 2 Centre for Advanced Medical Research and Training (CAMRET), Department of Medical Biochemistry, Usmanu Danfodiyo University Sokoto, Sokoto 840232, Nigeria; mustyimam@gmail.com

**Keywords:** ferrroptosis, lipid peroxidation, glutathione peroxidase 4, health-promoting

## Abstract

Natural bioactive compounds abundantly presented in foods and medicinal plants have recently received a remarkable attention because of their various biological activities and minimal toxicity. In recent years, many natural compounds appear to offer significant effects in the regulation of ferroptosis. Ferroptosis is the forefront of international scientific research which has been exponential growth since the term was coined. This type of regulated cell death is driven by iron-dependent phospholipid peroxidation. Recent studies have shown that numerous organ injuries and pathophysiological processes of many diseases are driven by ferroptosis, such as cancer, arteriosclerosis, neurodegenerative disease, diabetes, ischemia-reperfusion injury and acute renal failure. It is reported that the initiation and inhibition of ferroptosis plays a pivotal role in lipid peroxidation, organ damage, neurodegeneration and cancer growth and progression. Recently, many natural phytochemicals extracted from edible plants have been demonstrated to be novel ferroptosis regulators and have the potential to treat ferroptosis-related diseases. This review provides an updated overview on the role of natural bioactive compounds and the potential signaling pathways in the regulation of ferroptosis.

## 1. Introduction

Death of cells is an inevitable and crucial event that might be a way to maintain tissue homeostasis [1]. Regulated cell death (RCD) is a death process in living organisms which can be triggered by disruption of intercellular or extracellular environment [2]. Over the past decades, several forms of RCD have been discovered, such as apoptosis, necroptosis, ferroptosis and pyroptosis. Among them, the completely physiological forms of RCD such as apoptosis, necroptosis and pyroptosis are generally referred to as programmed cell death (PCD) [2]. Ferroptosis, a novel form of RCD, is initiated by oxidative perturbations of the intracellular micro-environment [2,3]. In fact, the term of ferroptosis was coined in 2012 to describe the type of cell death triggered by anticancer molecule erastin, which inhibited the activity of cystine-glutamate antiporter (system Xc^−^) and consequently resulted in the depletion of glutathione (GSH) and inactivation of the phospholipid peroxidase glutathione peroxidase 4 (GPX4) [4,5]. As a matter of fact, ferroptosis is triggered by the disruption of cellular redox homeostasis. Iron metabolism and lipid peroxidation is central mediators of ferroptotic process. Although ferroptosis is a recently recognized mode of cell death, it plays a crucial role in many human diseases and is identified as a potential therapeutic target [6]. According to recent studies, ferroptosis is closely linked to multiple physiological and pathological processes in humans and animals, including cancer, arteriosclerosis, ischemia-reperfusion injury, neurodegenerative diseases, and acute renal failure [7,8,9,10,11]. Therefore, exploring the molecular mechanisms and signaling pathways of ferroptosis and the targets of medical interventions may provide novel preventive and therapeutic strategies for many diseases.

It is reported that dysregulation of ferroptosis is closely connected with numerous physiological conditions and pathological stress [12]. The activation of ferroptosis might be a useful strategy for eliminating malignant cells and overcoming drug resistance in traditional cancer therapy [12]. In addition, ferroptosis can be a therapeutically target to ameliorate histopathological lesion of organs [13]. Based on the intensive study on ferroptosis, many clinical drugs and reagents have been demonstrated to be able to regulate ferroptosis. Chemotherapeutic agents such as sulfasalazine and cisplatin, targeted agents such as sorafenib and lapatinib, and antibiotics are proven as ferroptosis-inducers [14,15,16,17]. However, many of these pharmaceutical agents are associated with a series of undesirable side-effects, such as endocrine dysfunction, peripheral neuropathies, hepatic fibrosis, gastrointestinal bleeding and kidney failure [18]. Nature bioactive compounds are rich in plant foods such as fruits, vegetables, grains, seeds, herbs and spices, which possess protective or disease preventive properties. Accumulated evidence suggests that many bioactive compounds and their secondary metabolites exert good properties in regulating ferroptosis [12]. Ferroptosis is a two-edged sword. On one hand, it could induce the non-apoptotic destruction of certain tumor cells. On the other hand, it might cause organ damage [11,19,20]. The aim of this review was to provide the updated information on natural compounds that have promising regulatory effects on ferroptosis and their molecular targets and mechanisms.

## 2. An Overview of Ferroptosis

Cell fate determination largely depends on the oxidative stress in cells. Oxidative modification of lipids in membrane bilayers, especially lipid peroxidation, is a key regulator of cell fate. In fact, ferroptosis-like cell death has been observed for a long time. As early as 2001, Tan et al. discovered a unique oxidative stress-induced programmed cell death pathway which is entitled “oxytosis” [21]. Then, in 2003, Dolma et al. found a new small molecular, erastin, which initiated a nonapoptotic cell death process without classic morphological or biochemical features of apoptosis and the process could not be inhibited by caspase inhibitors [22]. Afterwards, researchers successively unveiled that this unique cell death mode could be suppressed by iron chelators and another compound RSL3 was identified being able to activate a similar pattern of non-apoptotic and iron-dependent cell death [23,24]. After these findings, ferroptosis was coined to depict this pattern of cell death in 2012 [4]. The cystine/GSH/GPX4 signaling axis is regarded as the predominant control system of ferroptosis. Distinctive lipid peroxidation and reactive oxygen species (ROS) overproduction are considered as the fatal factors. Some studies have discovered that ferroptosis is widely involved in the process of numerous diseases [13]. It is implied that ferroptosis is a pathophysiological process widely existing in organism rather than an organ-specific process. Meanwhile, multiple organelles such as mitochondria, endoplasmic reticulum, golgi apparatus and lysosomes are implicated in the process of iron metabolism and redox imbalance in ferroptosis [25]. It is reported that ferroptotic cell death can be excellently counteracted by lipophilic antioxidants (ferrostatin-1 and α-tocopherol), iron chelating agents, lipid peroxidation inhibitors and consumption of polyunsaturated fatty acids [3]. However, whether ferroptosis is strongly involved in more human diseases and more organelles, the precise mechanisms and biological functions of ferroptosis remain poorly understood. 

## 3. Mechanisms of Ferroptosis

**Figure 1:**
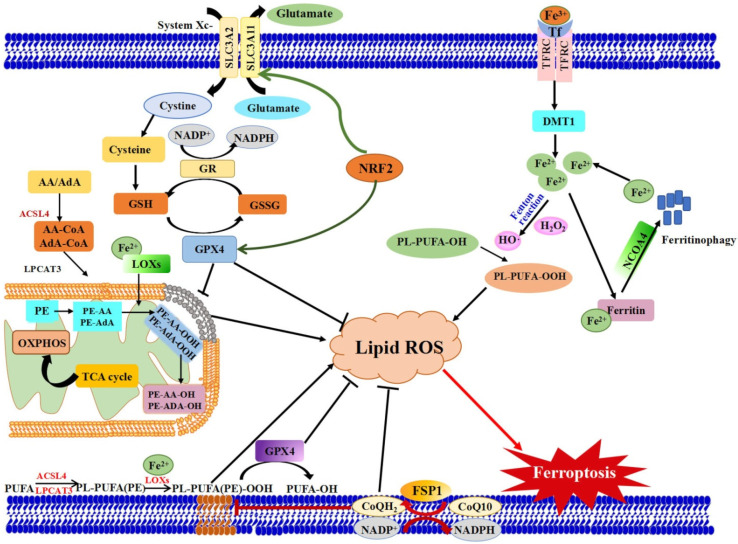
Schematic view of the molecular pathways of ferroptosis regulation. Three main metabolic pathways are GSH/GPX4 pathway, lipid peroxidation and iron metabolism pathways. Ferroptosis is initiated by the suppression of system Xc^−^ and depletion of GSH, or inhibition of GPX4, which results in cell death. Lipid ROS is in charge of the process of ferroptosis. The peroxidation of PUFAs is identified as a vital contributor. Excess iron is the basis for ferroptosis execution. In addition, the latest researches have revealed that the FSP1-CoQ10-NAD(P)H pathway with its unique mechanistic properties engages in ferroptosis. AA: Arachidonic acid, ACSL4: Acyl-CoA Synthetase Long Chain Family Member 4), AdA: Adrenoyl, DMT1: Divalent metal transporter 1, FSP1: Ferroptosis suppressor protein 1, FPN1: Ferroportin 1, GPX4: Glutathioneperoxidase 4, GSH: glutathione, GSSG: Oxidized GSH; LPCAT3: Lysophosphatidylcholine acyltransferase 3, lipoxygenases: LOXs, NCOA4: Nuclear receptor coactivator 4, GR: Glutathione reductase. PL: Phospholipids, Tf: Transferrin.

Ferroptosis is controlled and executed by an integrated cell signaling network. Researchers have revealed that amino acid and glutathione (GSH) metabolism, iron metabolism, lipid peroxidation, ferroptosis-suppressor-protein 1 (FSP1)-coenzyme Q10 (CoQ10) axis and other regulators are involved in the regulatory mechanisms of ferroptosis (Figure 1) [4,5,26,27].

### 3.1. Amino Acid and GSH Metabolism

Amino acid and GSH metabolism are tightly linked to the regulation of ferroptosis. The amino acid antiporter system Xc^−^ is consisted of solute carrier family 3 member 2 (SLC3A2) and the catalytic subunit solute carrier family 7 member 11 (SLC7A11) [28]. The system Xc^−^ takes charge of redox homeostasis by exchange of extracellular cystine and intracellular glutamate at the ratio of 1:1. The imported cystine can be promptly converted to cysteine and serves as substrate for GSH synthesis. Then, GPX4, a central regulator of ferroptosis, utilizes the antioxidant GSH to detoxify lipid peroxidation and modulate the initiation of ferroptosis [5]. Inhibiting system Xc^−^ with small molecular (e.g., erastin) can decrease the cellular uptake of cysteine, inhibit GSH synthesis and inactivate GPX4, which in turn, triggers excessive lipid peroxidation and ferroptotic cell death [5,29]. According to study, the alteration of GPX4 level and/or activity will immediately affect cell survival [30]. In vivo study has confirmed the core role of GPX4 in ferroptosis. Inactivation of GPX4 could cause severe acute renal failure in mice [11]. Acting as an essential cofactor of GPX4, GSH could reduce lipid peroxides, playing the ferroptosis-resistance activity [3]. The ferroptotic agonist (1S, 3R)-RSL3 can bind and irreversibly inactivate GPX4, leading to cell death [17]. Therefore, gene knockout and inactivation of GPX4 is vital way to induce ferroptosis. It is suggested that system Xc^−^-GSH-GPX4 may be effective therapeutic approach in organ damage mediated by ferroptosis.

### 3.2. Iron Metabolism

Iron is a redox-active metal. Excessive iron contributes to the execution of ferroptosis through inducing the production of ROS by Fenton chain reaction. Thus, the sensitivity of ferroptosis is closely associated with iron homeostasis including iron uptake, export, storage and turnover. Iron homeostasis is a complex process, which relies on coordination of multiple genes such as *IREB2* (iron responsive element binding protein 2), *FTH1* (ferritin heavy chain 1), *FTL* (ferritin light chain), *TF* (transferrin), *TFR1* (transferring receptor 1), *FPN* (ferropotein) and *DMT1* (divalent metal-ion transporter-1). These genes encoded proteins could affect ferroptosis trigger via regulating intracellular iron. A recent study found that Tf and TfR1, mainly responsible for intestinal iron absorption, play the critical role in ferroptotic cell death [23,31]. Elevated plasma level of TfR1 increased the amount of intracellular iron, which might promote ferroptosis, e.g., leading to auditory cortex neurodegeneration [32]. The redundant intracellular iron is stored in ferritin which is composed of FTL and FTH1. RSL3-sensitive cells transformed with oncogenic RAS significantly could increase cellular iron level via increasing *TfR1* and decreasing *FTL* and *FTH1* mRNA expression [23]. The selective cargo receptor-nuclear receptor coactivator 4 (NCOA4)-regulated degradation of ferritin (referred to as ferritinophagy) influences the availability of labile iron and stimulates erastin-induced ferroptosis [33,34,35]. The bromodomain protein BRD4 inhibitor (+)-JQ1 could induce ferroptosis via ferritinophagy and regulate ferroptosis-related genes in cancer cells [36]. IREB2 is a master regulator of iron metabolism. Silencing of *IREB2* by shRNA inhibits the sensitivity to erastin-induced ferroptosis in HT1080 cells [4]. Based on these findings, the cellular iron metabolism is indispensable for the induction of ferroptosis. Strategy targeting iron metabolism genes might be a promising method to treat the cancers which are drug-resistant or alleviate ferroptosis-induced diseases.

### 3.3. Lipid Metabolism

Lipid metabolism is closely associated with the process of ferroptosis. The accumulation of lipid peroxide could directly damage cellular and organelle membrane, initiating ferroptotic cell death. Polyunsaturated fatty acids (PUFAs) serve as the main substrates of lipid peroxidation during ferroptosis [37]. Thus, the extent of lipid peroxidation and the process of ferroptosis in cells largely depend on the content and localization of PUFAs. According to some reports, PUFA- phosphatidylethanolamines (PEs) esterified with arachidonoyl (AA) and adrenoyl (AdA) acyl chains are the key phospholipids in triggering ferroptosis [26,38]. The free AA/AdA could bind to coenzyme A (CoA) to form AA/AdA-CoA under the action of Acyl-CoA synthetase long-chain family member 4 (ACSL4), facilitating their esterification into phospholipids. Subsequently, lysophosphatidylcholine acyltransferase-3 (LPCAT3) catalyzes AA/AdA-CoA esterified into PEs and then the formed AA/AdA-PEs would be oxidized into lipid hydroperoxides by lipoxygenase (LOX), inducing ferroptosis [39]. Therefore, ACSL4 and LPCAT3, two enzymes involved in the biosynthesis and remodeling of PUFA-PEs in cellular membranes, are critical determinants of ferroptosis sensitivity [3]. Genetic disruption of *ACSL4* and *LPCAT3* depletes the substrates for lipid peroxidation and decreases ferroptosis sensitivity [38,39,40]. It is reported that cells treated with arachidonic acid or other PUFA are sensitized to ferroptosis [26]. Peroxidation of n-3 and n-6 PUFA exerted selective cytotoxic effects in acidic cancer cells, leading to ferroptosis-mediated antitumor effects [41]. In addition, LOXs also contribute to ferroptosis. The suppression of LOX by ferroptosis inhibitors, including vitamin E, zileuton and baicalein, can relieve ferroptosis [42,43,44]. However, some LOXs are essential for normal embryonic development in vertebrates. Knockdown of 12S-LOX in zeberafish could severely impair embryonic phenotype, characterized by abnormal brain, eyes, tails as well as yolk sac and pericardial edema [45]. It is suggested that lipid metabolism is required for both ferroptosis and normal physiological function. Abnormal lipid metabolism could be a crucial trigger for ferroptosis.

### 3.4. The FSP1-CoQ10 Axis

FSP1 was previously called apoptosis inducing factor mitochondria associated 2 (AIFM2). Recently, two momentous back-to-back research by Doll et al. and Bersuker et al. disclosed that FSP1 is a novel inhibitor of ferroptosis and could counteract ferroptosis independently even in the absence of GPX4 [46,47]. AIFM2 is proposed before the concept of ferroptosis, whose activation could suppress tumor proliferation and enhance apoptosis [48]. In human lung cancer cell lines, the activation of AIFM2 markedly contributed to cancer cells apoptosis undergoing exogenous toxicological stimulus [49]. Recently, Bersuker and his colleagues identified that FSP1 was a potent ferroptosis-resistance factor through using a synthetic lethal CRISPR-Cas9 knockout screen [47]. Almost simultaneously, Doll et al. also executed relevant studies on FSP1 [46]. By using an overexpression screen, their group demonstrated that FSP1 complemented the loss of GPX4 or inhibition of GPX4 in human cancer cells. The inhibition of ferroptosis by FSP1 was mediated by antioxidant CoQ10, while FSP1 could catalyze the regeneration of CoQ10 by NAD(P)H [46,47]. Therefore, FSP1-CoQ10-NAD(P)H axis is an independent parallel system, cooperating with GPX4 and GSH/GSSG to inhibit lipid peroxidation and control the process of ferroptosis. Both studies disclosed that the expression of FSP1 was negatively correlated with the ferroptosis sensitivity in many cancer cell lines. In conclusion, the discovery of FSP1 supplemented and perfected the ferrroptosis pathway. FSP1 can be used as a valuable biomarker of ferroptosis resistance. Exploiting FSP1 inhibitors can be effective strategy to overcome ferroptosis resistance in cancer cells.

### 3.5. Other Regulators

There are some regulators and key components that act vital roles in the ferroptosis cascade (Figure 1).

#### 3.5.1. Nrf2

The transcription factor Nrf2 is a key regulator that maintains cellular redox homeostasis and plays a critical role in mediating iron/heme metabolism [50]. It is reported that most of the transcription of ferroptosis-related genes are regulated by Nrf2. Both iron metabolism-related genes (*TFR1*, *FPN*, *FTH1* and *FTL*) and heme metabolism–related genes (*HO-1*, *SLC48A1*) are Nrf2 target genes [51]. Nrf2 activation enables decrease in the intracellular iron pool to restore iron homeostasis, restricts the production of reactive oxygen species (ROS) and upregulates SLC7A11. The expression of *SLC7A11* is positive correlation with activity of system Xc^−^. Upregulation of *SLC7A11* expression at the transcription level could enhance cystine uptake and consequently inhibit ferroptosis [52] (31,908,494). Therefore, Nrf2 is considered to be a negative regulator of ferroptosis. As expected, activation of Nrf2 pathways confers resistance to GPX4 inhibitor induced ferroptosis in cancer cells [53]. These results indicate that targeting Nrf2 or its downstream targets could be a dependable approach to regulate ferroptosis. Paradoxically, ferroptosis activators erastin and sorafenib can enhance Nrf2 expression in HCC cells, whereas Nrf2 activation could negatively regulate ferroptosis [53,54]. Thus, the modification of Nrf2 signaling can be a good strategy for disease intervention via regulating ferroptosis process.

#### 3.5.2. P53

**Figure 2:**
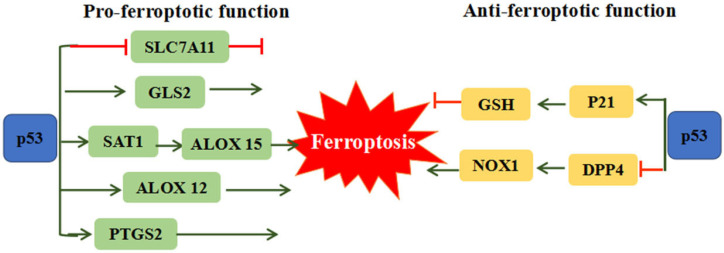
The dual function of p53 on ferroptosis. P53 can promote ferroptosis through regulating SLC7A11, GLS2, SAT1/ALOX15, ALOX12 and PTGS2. Meanwhile, p53 also could restrain ferroptosis via the mediation of p21 and DPP4. ALOX12: Arachidonate 12-Lipoxygenase, ALOX15: Arachidonate 15-Lipoxygenase, DPP4: Dipeptidyl peptidase 4, GLS2: Glutaminase 2, SLC7A11: Solute carrier family 7 member 11, SAT1: Spermidine/spermine N1-acetyltransferase 1.

The p53 tumor suppressor is distinguished as the most frequently mutated gene in human cancer, which is involved in cell-cycle arrest, cellular senescence and apoptosis [55]. Recent studies revealed the dual effects of p53 on ferroptosis (Figure 2). On one hand p53 can remarkably stimulate ferroptosis through downregulation of SLC7A11 expression or upregulation of spermidine/spermine N’-acetyltransferase 1 (SAT1) and glutaminase 2 (GLS2) expression [56]. The inhibition of SLC7A11 by p53 could facilitate cells to ferroptosis via suppressing cysteine uptake. *SAT1* and *GSL2* genes are both transcriptional target of p53. SAT1 activation can promote the expression of ALOX 15, induce lipid peroxidation and sensitize cells to undergo ferroptosis [57]. GLS2 takes part in the process of ferroptosis via promoting cellular ROS production and decreasing GSH [58]. ALOX12 (arachidonate 12-lipooxygenase), a member of a nonheme lipoxygenase family of dioxygenases, has been regarded as an essential factor of p53-mediated ferroptotic responses, but not required for GPX4 and ACSL4-mediated ferroptosis [59]. On the other hand, p53 can restrain ferroptosis through directly inhibiting dipeptidyl peptidase 4 (DPP4) activities or through inducing cyclin dependent kinase inhibitor 1A/p21 (CDKN1A/p21) in a transcription-independent manner. Absence of p53 could facilitate DPP4 to interact with NADPH oxidase 1 (NOX1) and form NOX1-DPP4 complex, triggering lipid peroxidation and ferroptosis. In human colorectal cancer, p53 could inhibit ferroptosis by transforming DPP4 from plasma membrane to nucleus to form DPP4-p53 complex, resulting in the dissociation of NOX1 and suppressing lipid peroxidation [60]. The p53 transcriptional target CDKN1A (encoding p21) is an important regulator of p53-dependent cell-cycle arrest, which is required for wild-type p53 stabilization. The p53-p21 transcriptional axis can suppress system Xc^−^ activity and simultaneously decline sensitivity to ferroptosis [61]. It is proposed that the converse effect of p53 on ferroptosis may due to different cell types.

#### 3.5.3. Heme Oxygenase (HO)-1

HO-1 is the first rate-limiting enzyme in the breakdown of heme, in converting heme into biliverdin, ferrous iron and carbon monoxide. As HO-1 is one of Nrf2 target genes, induction of HO-1 exerts important cytoprotective and antioxidant properties [62]. Study has demonstrated the important antiferroptotic role of HO-1 in renal proximal tubule cells. The absence of HO-1 significantly increased erastin- and RSL3-induced cell death in renal proximal tubule cells isolated from HO-1^−/−^ mice [63]. Similar results reported the negative function of HO-1 in erastin- and sosrafenib-induced hepatocellular carcinoma ferroptosis as knockdown of HO-1 [7]. Intriguingly, the regulatory effect of HO-1 on ferroptosis is complicated. Upregulated HO-1 activity could stimulate heme degradation and increase cellular iron level. Emerging evidence has revealed that HO-1 could induce ferroptosis via the induction of iron overload and excessive accumulation of ROS and lipid peroxidation [64,65]. The overexpression of HO-1 augments erastin-induced ferroptotic cell death in HT1080 cells [64]. Therefore, HO-1 has dual effects in ferroptosis process.

## 4. Natural Bioactive Compounds as Ferroptosis Regulators

Natural bioactive compounds are potentially beneficial phytochemicals found in plant foods and have been reported to possess multiple health-promoting bioactivities, including anticancer, antioxidant, immunomodulation, antibacterial and antiparasitic activities [66]. These bioactive molecules have raised great interest for the prevention and treatment of numerous disorders of metabolism based on their bioactive properties [67]. However, the potential mechanisms and targeting signaling pathways of them are not entirely known.

Since ferroptosis is usually accompanied by iron overload and cellular ROS accumulation, the lipophilic antioxidants and iron chelators are potential candidate inhibitors of ferroptosis. Thus, many bioactive compounds have the potential to regulate ferroptosis through inhibiting lipid peroxidation and iron overload. Recently, numerous studies have focused on discovering novel bioactive compounds as ferroptosis regulators and related molecular mechanisms in the treatment of many diseases and drug resistance.

### 4.1. Apigenin

Apigenin is one of the most abundant natural flavonoids found in a range of dietary plant foods, including fruits, vegetables, wheat sprouts and some seasonings [68]. It has attracted extensive attention due to its valuable health-promoting function and its notable cytotoxic effect on cancer cells [69]. As tumor suppressor, apigenin has the ability to inhibit tumor growth via the promotion of cell-cycle arrest, apoptosis and autophagy, preventing tumor cell proliferation, migration and invasion [69]. In Adham’s study, apigenin triggered cell death through ferroptosis in NCI-H929 cells [70]. However, apigenin has dual characters, which can inhibit ferroptosis in some case. It was reported that apigenin could relieve the myeloperoxidase-mediated oxidative stress, could serve as an efficient activator for GPX4 and SIRT1 and could down-regulate level of Ac-p53, thereby inhibiting ferroptosis in the brain of kainic acid-induced epileptic mice [71]. These studies provide a versatile strategy for the discovery of small molecular agents for ferroptosis-related diseases prevention and treatments.

### 4.2. Artemisinin and Its Derivatives

*Artemisia* plants, known as sweet wormwood, have attracted tremendous attention due to the excellent biological activities and minimum adverse effects. *Artemisia* species display high food value, which are extensively used in countries of Europe, Asia and North America [72]. Many *Artemisia* species are applied to prepare beverages, flavorings and dietary supplements [72]. Artemisinin is an effective antimalarial drug originated from *Artemisia* plants. In 2015, the Nobel Prize for Medicine was awarded to Youyou Tu for the drug’s discovery and efficacy. Artemisinin, referred to as the most promising antimalarial drug, also has the potential to treat cancer, inflammations, viral infections, hepatitis and headaches [73]. At present, numerous derivatives of artemisinin such as esters, ethers, dimers, trimers, and tetramers have been synthesized and expected to become efficient drug candidates [74]. Some researchers have found that these compounds can produce good curative effects on many diseases such as inflammation, infection, cancer, anthelmintic and fibrosis [75,76,77,78]. Recently, artemisinin and its derivatives were identified as efficient activators of ferroptosis in many different types of cancer in vitro and in vivo [79]. The molecular mechanisms relate to down-regulation of GPX4 expression, modulation of cellular iron homeostasis [80], the stimulation of intracellular ROS production, the lysosomal degradation of ferritin, regulation of system Xc^−^/GPX4 axis, Nrf2-ARE pathway inhibition in resistant head and neck cancer cells [81] and the activation of ATF4-CHOP-CHAC1 pathway in Burkitt’s lymphoma [82]. In addition, researchers found that dihydroartemisinin or artemisinin-induced ferroptosis could be disturbed by GRP78 in glioma cells and *KRAS* mutant pancreatic cancer cells [79,83,84]. Therefore, these studies shed light on the molecular mechanism of artemisinin and its derivatives in ferroptosis, which could be used as cancer therapies with broader application attributable to the induction of ferroptosis.

### 4.3. Baicalein

Baicalein (5,6,7-trihydroxy-2-phenyl-4H-1-benzopyran-4-one, C_15_H_10_O_5_) is a flavonoid compound mainly derived from the root of *Scutellaria baicalensis* Georgi. In Eastern and Western cultures, baicalein is extensively applied in the production of dietary food supplement [85]. In addition, it has been widely used for the treatment of inflammation, hypertension, cardiovascular disease, diabetes, bacterial infection, cancer and neurotoxicity [86,87,88]. Recently, Xie et al. firstly identified baicalein as a ferroptosis inhibitor. The anti-ferroptosis activity of baicalein was referred to the modification of iron accumulation, glutathione depletion and lipid peroxidation [89]. Li et al. found that baicalein could alleviate ferric ammonium citrate-induced neuronal damage by inhibiting ferroptosis [43]. The addition of baicalein, a selective 12/15-LOX inhibitor, significantly decreased RSL3-stimulated ROS generation and lipid peroxidation, alleviated cell death in both Jurkat and Molt-4 cells [90]. Another important reason may be that baicalein could modulate body’s iron homeostasis through strongly binding the ferrous ion and inhibiting iron-promoted fenton chemical reaction [91]. Previously, Gao et al. obtained similar results that baicalein bound to microsomal membranes inhibited lipid peroxidant by forming iron-baicalein complex [92]. Thus, baicalein can be used as a natural ferroptosis inhibitor via decreasing ROS generation, modulation of iron homeostasis, binding the ferrous iron and suppresses degradation of GPX4. Baicalein would be a potential therapeutic agent for ferroptosis-associated tissue injury.

### 4.4. Brusatol

The natural product isolated from the fruit of *Brucea javanica* (L.) Merr., brusatol, is an inhibitor of Nrf2. The recent investigations have reported that brusatol is a promising therapeutic agent for human malignancies, such as leukemia, lung cancer, pancreatic cancer and brain tumor [93,94]. In ovarian cancer cells, iron overload was associated with cisplatin resistance. Brusatol remarkably overcame cisplatin resistance and enhanced the antitumor effect via reducing intracellular iron [95]. The treatment with the combination of brusatol and erastin showed better therapeutic outcome against human non-small-cell lung carcinoma model than single treatment via the activation of FOCAD-FDK (focal adhesion kinase) pathway [96]. Ge et al. revealed that ferroptosis plays a vital role in the spinal cord injury process. Zinc could alleviate ferroptosis and improve functional recovery from contusion spinal cord injury through activation of Nrf2/GPX4 pathway, whereas this effect was reversed by the Nrf2 inhibitor brusatol. It follows that brusatol could regulate ferroptosis process through blocking Nrf2 signaling [97].

### 4.5. Curcumin

Curcumin ([1,7-bis(4-hydroxy-3-methoxypheny 1)-l,6-heptadiene-3,5-dione]), a phenolic compound from turmeric (*Curcuma longa*), has been used extensively as a kind of food additive for centuries [98]. It can give yellowish color and distinct taste to foods. In recent decades, research shows that curcumin has many biological activities such as antioxidant, anti-inflammatory and antitumor properties [99]. Recent research from Guerrero-Hue et al. showed that curcumin could reduce the functional and structural injury in rhabdomyolysis-mediated renal damage through decreasing lipid peroxidation and ferroptosis. These protective effects were mediated by HO-1 [100]. In murine MIN6 cells, curcumin exerted its protective effects against iron toxicity and erastin-induced ferroptosis via mitigating iron overload and lipid peroxidation [101]. Many years ago, scientists had discovered that curcumin is a strong chelator of iron under neutral to slightly acidic conditions [102]. The iron-binding capacity may contribute to its anti- lipid peroxidation and anti-ferroptosis activities. Conversely, curcumin triggered ferroptosis in breast cancer cells through causing iron accumulation and upregulation of HO-1 [103]. The curcumin analog ALZ003 treatment disrupted GPX4-mediated redox homeostasis and induced ferroptosis in glioblastoma cell [104]. Therefore, curcumin may exert its properties through regulating the process of ferroptosis.

### 4.6. Epigallocatechin-3-Gallate (EGCG)

EGCG is a type of plant-based compound called catechin. It is the most abundant and active compound in green tea leaves. Furthermore, apples, blackberries, carob flour and many other foods also naturally contain EGCG. Extensive research has shown that it has significant antioxidant, anti-inflammation, anticarcinogenic, antimicrobial, anti-infection and neuroprotective properties and has therapeutic potential against various human diseases [105,106,107]. By consuming foods that contain this substance, people may be able to improve health and reduce incidence of disease. Several studies identified EGCG as a novel ferroptosis inhibitor. Kose et al. discovered that EGCG protected pancreatic cells against erastin-induced ferroptosis through chelating iron and preventing GSH depletion and lipid peroxidation [101]. Inhibition of ferroptosis by EGCG reduced oxidative stress and promoted recovery in spinal cord transection rats [108]. In addition, Nrf2 and its downstream targets comprising antioxidant proteins SLC7A11, HO-1 and GPX4 are essential for the radioprotective effects of EGCG in HIEC cells [109]. Collectively, EGCG could modulate ferroptosis-related signaling receptor and signaling pathway to play its pharmacological role through above mechanisms.

### 4.7. Erianin

*Dendrobium* genus is a traditional medicinal and edible food, which mainly consists of *Dendrobium nobile* Lindl., *Dendrobium candidum* Wall., *Dendrobium fimbriatum* Hook, and *Dendrobium chrysotoxum* Lindl. *Dendrobium* genus contains many nutrients and biologically active metabolites, attracting extensively attention in scientific community [110]. Erianin [2-Methoxy-5-(2-(3,4,5-trimethoxyphenyl)-ethyl)-phenol], a dietary compound derived from *Dendrobium chrysotoxum Lindl*, has been discovered to possess antitumor, antipyretic and analgesic effects in traditional Chinese medicine [111,112,113]. In recent years, many studies have proved that erianin has strong anticancer activities in various cancer cells [114,115,116]. Erianin could induce cell apoptosis, autophagy, anti-angiogenesis and cell cycle arrest, and inhibit tumor cell metastasis via different signaling pathways [111]. A recent study from Chen et al. demonstrated for the first time that erianin induced ferroptotic cell death in lung cancer cells through Ca^2+^/CaM signaling pathway, inhibiting cell migration and proliferation [117]. Thus, erianin might be a novel ferroptosis stimulator and be promising to be a potential natural phytochemical for cancer prevention.

### 4.8. Piperlongumine

Piperlongumine is an amide alkaloid derived from the edible long piper plants. The fruits of piper, which contain piperlongumine, are widely used as a spice, in pickles, preservatives, foods, beverages, liquors and medicines. Research shows that piperlongumine has exhibited cytotoxicity against a broad spectrum of human cancer cell lines and demonstrated antitumor activity in rodents [118]. Therefore, piperlongumine is a promising antitumor agent. Study has reported that piperlongumine could kill cancer cells through induction of ROS production and GSH depletion in cancer cells (EJ, MDAMB231, U2OS and MDAMB435), but did not increase ROS level in normal cells [119]. Yamaguchi et al. reported that piperlongumine induced human pancreatic cancer cell line death by dramatically increasing the intracellular ROS level and inhibited GSH activity. This effect was inhibited by ferroptosis inhibitors and iron chelators, but not apoptosis or necrosis inhibitors, which suggested that piperlongumine could induce cell death via ferroptosis [120]. This implies that piperlongumine may be a candidate for cancer therapy by inducing ferroptosis.

### 4.9. Quercetin

Quercetin (3,3′,4′,5,7-pentahydroxyflavone) is one of the most widely distributed flavonoids found in vegetables and fruits. Some of the most commonly consumed quercetin foods include apples, asparagus, berries, onions, red wine, tea, beans and tomatoes. Quercetin possesses a wide spectrum of noticeable biological activities, thus suggesting a role in disease prevention and health promotion [121]. Li and her colleagues found that ferroptosis contributed the dysfunction of pancreatic β cells. Quercetin could inhibit iron deposition, alleviate lipid peroxide and restore VDAC2 expression, exerting beneficial effects on pancreatic β cells in type 2 diabetes through anti-ferroptosis [122]. In erastin-treated bone marrow-derived mesenchymal stem cell (bmMSCs) model, quercetin protected against erastin-induced ferroptosis through antioxidant pathway [123]. In addition, quercetin possesses a protective role on acute kidney injury induced by ischemia-reperfusion or folic acid. The mechanism is that quercetin could significantly inhibit the expression of transcription factor 3 (ATF3) and further block the downstream pathway of ferroptosis [124]. Conversely, quercetin (12.5~100 μM) can also promote ferroptosis-dependent cell death in various cancer cells through stimulating ferritin degradation and free iron release and inducing lipid peroxidation [125]. Collectively, these studies identified quercetin as a ferroptosis regulator and provided the novel strategies in management of diseases related to ferroptosis.

### 4.10. Sterubin

The flavanone sterubin is the active component isolated from the plant Yerba santa (*Eriodictyon californicum* (Hook. and Arn.) Torr.). Yerba santa is a plant that has been brewed into a tea and used as a bitter flavoring additive to foods historically by Native Americans [126]. Previous studies have demonstrated that sterubin extracted from Yerba santa exhibited strong neuroprotective properties and held notable potential as a neuroprotectant [127,128]. Recently, scientists found that sterubin is an effective iron chelator, which could inhibit ferroptosis in a dose-dependent way through increasing GSH, decreasing ROS levels and activating Nrf2/ATF4 signaling pathway in glutamate-treated HT22 cells [129]. Flavanone sterubin as a potent neuroprotective natural compound has potential to be novel therapeutic agent for the treatment of neurodegenerative diseases.

### 4.11. Trigonelline

Trigonelline, one of the alkaloids contained in coffee beans and fenugreek seeds, present in several species of fruits and seeds. It is conducive indirectly to the formation of favorable flavor substance during coffee roasting. Due to its pharmacological value and low toxicity, the favored characteristics, trigonelline has attracted increasing attention in recent years. Numerous biological activities have been reported that trigonelline is beneficial in the prevention and treatment of diabetes, hyperlipidemia, nervous and hormonal disorders, and cancers [130]. It is found that trigonelline is an efficient Nrf2 inhibitor and is capable of blocking Nrf2/ARE pathway [131] According to two studies of Dr. Jong-Lyel Roh, trigonelline reversed RSL3 and cisplatin-induced resistance to ferroptosis in head and neck cancer cells via inhibition of the Nrf2 system [81,132]. In addition, the alkaloid trigonelline also blocked the expression of Nrf2 target gene NQO1, HO-1 and FTH1; suppression of Nrf2 activation in HCC cells increased the anticancer activity of erastin and sorafenib via ferroptosis [7]. Therefore, we speculate that the biological activities of trigonelline partially might rely on its ferroptosis-regulating properties. (The above-mentioned natural compounds were summarized in Table 1)

### 4.12. Other Natural Compounds

To date, dozens of bioactive natural compounds have been reported to possess the regulatory effects on ferroptosis process. In fact, previous studies have found some natural compounds exerted their effects through modulating GPX4 activity or chelating iron before “ferroptosis” being coined, such as salsolinol and β-phenylethyl isothiocyanate. Many kinds of natural compounds which could induce or inhibit ferroptosis are list in Table 2 and Table 3, respectively.

Taken together, over the past of several years tremendous efforts have been made to explore the regulatory mechanisms of natural agents on ferroptosis. Natural compounds mainly mediate ferroptosis through regulating system Xc^−^, GPX4 activity, lipid metabolic balance and iron homeostasis. Intriguingly, certain kinds of natural compounds exhibit two opposing effects on ferroptosis, initiating and terminating the process of ferroptosis. Hence, natural bioactive compounds are magic compounds and have the potential to be developed as therapeutic agents against diseases related to ferroptosis.

## 5. Discussion and Prospects

For the past decades, people are more interested in consuming natural compounds to improve the health and welfare of mankind. Natural compounds have been extensively reported to possess regulatory effects on ferroptosis. It is interesting that the regulatory effects and pathways of different natural compounds on ferroptosis are inconsistent. Artemisinin and its derivatives, brusatol, erianin, piperlongumine andtrigonelline could induce ferroptosis, whereas baicalein, EGCG and galangin significantly inhibit ferrroptosis. Therefore, natural compounds have promising potential to be developed as nutraceutical, functional foods and therapeutic agents for disorders that accompanied with ferroptosis. However, there are some factors that limit their use in practice are the poor aqueous solubility, high lipophilicity and abundant first pass metabolism. Therefore, it is better to pair phytochemicals-rich meals with foods containing fat. As for dietary supplements and natural compound fortified foods, the modern drug delivery strategies such as nanotechnology, polymeric micelles, liposomes and co-crystals can well surpass these limitations. Given the high safety of natural compounds, eating more foods rich in natural active compounds or fortified with natural compounds may exert promising preventive strategies against ferroptosis-related diseases and improve human health.

However, it is still unclear whether ferroptosis has hormesis feature. It is known that hormesis is considered as a mechanistic approach to explain the effects of herbal treatments in traditional Chinese medicine [164,165]. Some of ferroptosis inhibitors such as natural compounds curcumin and baicalein have hormetic properties, which can induce lipid ROS accumulation or ferroptosis in cancer therapy [165,166]. In addition, the pro- or anti-ferroptosis effects of natural compounds may relate to the types of cells. Quercetin, apigenin, sulforaphane and curcumin could trigger ferroptosis in cancer cells, while suppressed ferroptosis to protect against pancreatic β cells dysfunction, epilepsy and renal damage. Therefore, it is essential to take special precaution when applying natural compounds as ferroptosis inhibitors or initiators.

Moreover, the obtained cell-based data and animal-based data concerned with the connection between ferroptosis and various diseases should be considered seriously because the conditions of cellular experiments are widely divergent with in vivo conditions, and the animal experiments are distinct from clinical trial. Up to now, no natural compounds of ferroptosis inhibitors or initiators have been applied in clinical trial to treat related illnesses (please take a look at www.clinicaltrials.gov for details, Retrieved 18 October 2021). In the following study, more preclinical trials of natural bioactive compounds and the explicit molecular mechanisms in regulating ferroptotic cell death are essential. As an aside, it will be complicated and challenging to investigate the potency of the synergy and interactions of combined bioactive natural compounds and therapeutic agents in modulating ferroptosis-related diseases. To maximize the potential of natural compounds for application in ferroptosis-related diseases and discovery of more functional foods, there is an urgent need for large-scale and multicenter collaborative studies. In summary, this review addressed the function and signaling pathway of natural products involved in the regulation of ferroptosis, which provided an update on what is currently demonstrated in this field and a new thought to unearth more novel bioactivities of natural products.

## Figures and Tables

**Table 1:** Representative natural bioactive compounds as ferroptosis regulators.

Natural Compounds	Function	Model	Mechanism in Ferroptotic Regulation	Reference(s)
Apigenin	Induce ferroptosis	NCI-H929 cells	Suppression of iNOS and COX-2 expression	[70]
	Inhibit ferroptosis	Epileptic mice and SH-SY5Y cells	Relieved oxidative stress, upregulated GPX4, SIRT1 and GSH expression	[71]
Artemisinin and its derivatives	Induce ferroptosis	Cancer cell lines, mice	Decreased GPX4 expression and GSH levels, increased lipid ROS and disturbed iron homeostasis	[79,80,81,82,83,84]
Baicalein	Inhibit ferroptosis	acute lymphoblastic leukemia cells, HT22	Inhibited lipid peroxidation and Fenton reaction, decreased 4-HNE, increased GPX4 and GSH levels	[43,90,91,92]
Brusatol	Induce ferroptosis	ovarian cancer cells, NSCLC cell lines	Inhibited Nrf2	[95,96,97]
Curcumin	Inhibit ferroptosis	Mice MIN6 cells,	Mitigated iron overload and lipid peroxidation, actived HO-1	[100,101,102]
	Induce ferroptosis	breast cancer cells	Caused iron accumulation, disrupted GPX4-mediated redox homeostasis	[103,104]
EGCG	Inhibit ferroptosis	MIN6 cells, HIEC cells, cerebellar granule neurons, rats	Chelated iron, prevented GSH depletion and lipid peroxidation, downregulated levels of ACSL4, COX2, NOX1 and PTGS2 and upregulated FTH1 and GPX4 expression	[101,108,109]
Erianin	Induce ferroptosis	Lung cancer cells	Induced ROS accumulation, lipid peroxidation and GSH depletion, actived Ca^2+^/CaM signaling	[117]
Piperlongumine	Induce ferroptosis	Pancreatic cancer cell	Increased intracellular ROS and GSH depletion	[120]
Quercetin	Inhibit ferroptosis	Pancreatic β cells, bmMSCs, NRK-52E cells and HK-2 cells	Inhibited iron deposition, MDA and lipid ROS, alleviated lipid peroxide	[122,123,124]
	Induce ferroptosis	cancer cell lines	Induced ROS and lipid peroxidation, released free iron	[125]
Sterubin	Inhibit ferroptosis	HT22 cells	Increased GSH, decrease ROS and activated Nrf2/ATF4 signaling pathway	[129]
Trigonelline	Induce ferroptosis	head and neck cancer cell, mice	Blocked Nrf2/ARE pathway	[81,132]

**Table 2:** The inductive effects and mechanisms of natural compounds on ferroptosis.

Natural Compounds	Model	Mechanism in Ferroptotic Regulation	Reference(s)
Albiziabioside A	HCT116 cells	Inhibited GPX4 expression and induced MDA level	[133]
Amentoflavone	U251 and U373 cell lines	Upregulated levels of iron, MDA, GSH and lipid ROS, downregulated MDA level, activated AMPK- mTOR pathway	[134]
Ardisiacrispin B	CCRF-CEM cells	Increased ROS production	[135]
Aridanin	CCRF-CEM leukemia cells	Increased ROS	[136]
Beta-elemene	HCT116 and Lovo	Induced GSH depletion and lipid peroxidation, upregulated HO-1 and transferrin, and downregulated protein expression of GPX4, SLC7A11, FTH1, glutaminase and SLC40A1	[137]
Beta-phenylethyl isothiocyanate	T72Ras cells	Inhibited GPX4 activity and caused severe ROS accumulation	[138]
Bromelain	Human colorectal cancercells	Induced ROS and acyl-CoA synthetase long chain family member 4	[139]
Cotylenin A	MIAPaCa-2, PANC-1, CFPAC-1	Triggered ROS accumulation	[140]
Crassin	MDA-MB-231 and 4T1	Induced cytostasis downstream of ROS activation	[141]
Dihydroisotanshinone I	MCF-7 cells and MDA-MB-231 cells	Repressed the protein expression of GPX4, increased the MDA level, decreased the GSH/GSSG ratio	[142]
Epunctanone	Cancer cell lines	Increased ROS production	[143]
Formosanin C	human HCC cell lines Hep3B and HepG2	Enhanced lipid ROS formation and NCOA4 level, and reduced FTH1 levels, inducing ferritinophagy	[144]
Gallic acid	MDA-MB-231 and A375	Increased ROS production and reduced GPX4 activity	[145,146]
6-Gingerol	A549 cells and mice	Increased ROS and iron concentration, promoted the expression of Beclin-1, LC3 I, LC3 II, NCOA4 and TfR1, down-regulated expression of USP14, FTH1, GPX4 and ATF4	[147]
Oridonin	TE1 cells	Reduced the value of intracellular GSH/GSSG, decreased GPX4 and inhibited the gamma-glutamyl cycle	[148]
Oleanolic acid	hela cells and tumor-bearing mice	Increased the oxidative stress level and Fe2+ content, activated ferroptosis by promoting ACSL4 expression	[149]
Ruscogenin	Pancreatic cancer cells	Increased ROS production and intracellular ferrous irons, regualated the levels of transferrin and ferroportin	[150]
Salsolinol	PC12 cells	Lowered the intracellular GSH content, which was restored by iron chelator	[151]
Sulforaphane	Small-cell lung cancer cell lines	Decrease mRNA and protein expression levels of SLC7A11, increase levels of Fe^2+^ and ROS	[152]
Talaroconvolutin A	Colorectal cancer cells	Increased ROS, downregulated expression of SLC7A11 and upregulated arachidonate lipoxygenase 3	[153]
Ungeremine	CCRF-CEM cells	Increased ROS levels and integrity of mitochondrial membrane	[154]

**Table 3:** The inhibitory effects and mechanisms of natural compounds on ferroptosis.

Natural Compounds	Model	Mechanism in Ferroptotic Regulation	Reference(s)
Artepillin C	HT22	Attenuated ROS mitochondrial superoxide anion production and increased intracellular Ca^2+^	[155]
Butein and (S)-butin	BMSCs	scavenged LOO • radicals and inhibited LPO	[156]
Cullen corylifolium psoralidin	HT22 cells	Inhibitory affinity for 5-LOX and Keap1-Nrf2 protein-protein interactions	[157]
Galangin	gerbils	Reduced the levels of lipid peroxide in the brains, increased expression of SLC7A11 and GPX4	[158]
Kaempferol	Mice, HT22 cells	Inhibited ROS production, activated Nrf2/SLC7A11/GPX4 axis	[155,159]
Morachalcone D and E	HT22 cells	Increased expression of GPx4, CAT, SOD2, Nrf2, HMOX1 and SLC7A11	[160]
Nuciferine	HK-2 and HEK293T, mice	Mitigated iron accumulation and lipid peroxidation, increased the expression of the GPX4, SLC7A11, FSP1 mRNA and proteins	[161]
Puerarin	H9c2 myocytes and rats	Alleviated iron overload and lipid Peroxidation, reduced NOX4 expression.	[162]
Sulforaphane	C2C12 myoblasts and KIKO mice	Inhibit lipid peroxides, activate NRF2 and GPX4	[163]

## Data Availability

Not applicable.
